# Is geriatric medicine teaching homogeneous? The analysis of geriatric medicine courses at Polish undergraduate medical programmes

**DOI:** 10.1007/s41999-024-01004-y

**Published:** 2024-06-19

**Authors:** Robert Kupis, Ian Perera, Tomasz Targowski, Jerzy Gąsowski, Karolina Piotrowicz

**Affiliations:** 1 Department of Medical Education, Centre of Innovative Medical Education, Jagiellonian University Medical College, Kraków, Poland; 2 Department of Internal Medicine and Gerontology, Faculty of Medicine, Jagiellonian University Medical College, Kraków, Poland; 3 Department of Geriatrics, National Institute of Geriatrics, Rheumatology and Rehabilitation, Warsaw, Poland

**Keywords:** Medical education, Geriatric medicine, Undergraduate course, Medical degree, Poland

## Abstract

**Purpose:**

We aimed to analyse the characteristics of geriatric courses offered to undergraduate medical students in higher educational institutions (HEIs) in Poland.

**Methods:**

We searched the official websites of the HEIs offering the medical degree programmes and directly contacted the dean’s offices and HEIs representatives to retrieve the relevant information. The documents were analysed for course content, teaching methods, duration, and recommended texts. We also checked the obtained curricula for the reference to of the learning objectives related to geriatric medicine, selected from the currently endorsed Polish educational standards (ES) provided by the Ministry of Science and Higher Education.

**Results:**

Geriatric medicine courses were obligatory at all included HEIs (*n* = 19), but the courses differed in structure and content. The courses varied in duration from 11 to 60 h and were primarily lecture based. Simulation was utilized at only one HEI and e-learning at two institutions. Out of 315 learning objectives, we acknowledged only 9 as geriatric. They were not always found in all curricula. Two HEIs included self-described learning objectives in their curricula. Across all HEIs, a total of 29 recommended texts (published between 1995 and 2021) were identified, including 2 English-language texts.

**Conclusion:**

Geriatric medicine was a mandatory subject for medical students of the included HEIs. However, there was a lack of uniformity in the offered courses. This leaves room for the development of a unified undergraduate geriatrics curriculum to effectively address diverse geriatric issues across Europe. The importance of this matter is highlighted by demographic trends and workforce challenges.

**Supplementary Information:**

The online version contains supplementary material available at 10.1007/s41999-024-01004-y.

## Key summary points

**Aim:**

To perform a deepened analysis of the contents of Polish undergraduate courses in geriatric medicine in Poland.

**Findings:**

Polish courses in geriatric medicine are heterogeneous, even though they are based on a common national educational standard provided by the Ministry of Higher Education and Science.

**Message:**

Geriatric medicine courses should be unified and modernised to adapt the teaching methods to the requirements of students and current trends in medical education, and to better prepare the alumni to face the challenging demographic predictions.

**Supplementary Information:**

The online version contains supplementary material available at 10.1007/s41999-024-01004-y.

## Introduction

To become a physician in Poland, it is mandatory to complete a medical degree (MD) programme offered by an accredited university or other form of higher education institution (HEI). Currently, there are 38 HEIs in Poland that offer MD in Polish. Importantly, 14 of them have recently started MDs and the 2023/2024 academic year is their first year of operations. Prior to 2015 there were 12 universities, including 9 medical universities supervised by the Ministry of Health and 3 universities with faculties of medicine (Jagiellonian University in Kraków, Nicolaus Copernicus University in Toruń, University of Warmia and Mazury in Olsztyn) supervised by the Ministry of Higher Education. Since 2015, new MDs have been launched at different HEIs across the country. The process has been met with criticism from many stakeholders, including policymakers, academics, and the medical community, including representatives of the Supreme Medical Chamber [1–4].

The framework for the programmes and curricula is regulated by the educational standards (ES) issued by the Minister of Science and Higher Education. Currently, all courses follow the ES published in 2019 [5] or the previous one, issued in 2012 [6]. In September 2023, the newest ES were published [7] and HEIs need to adapt their programmes in accordance with these updates. It should provide valuable opportunity for all institutions to modernise their programmes, including teaching methods, time devoted to each discipline, and materials. On the other hand, it may become an additional burden to those who are not keen to overhaul the established teaching norms.

The ES establishes the basis of the overall medical programme, but all details are determined at the discretion of a given HEI’s authorities. The standards indicate the minimum total number of hours and ECTS points assigned for the programme, and the minimum duration of the programme, all of which must be met by the HEIs and their teaching staff. The most crucial part of the ES are learning objectives. According to the legal regulations, every MD programme may be organised differently, but they must enable all students to obtain comparable outcomes. These outcomes are defined by the learning objectives, which are divided into a few groups with a minimum amount of time assigned.

**Fig. 1:**
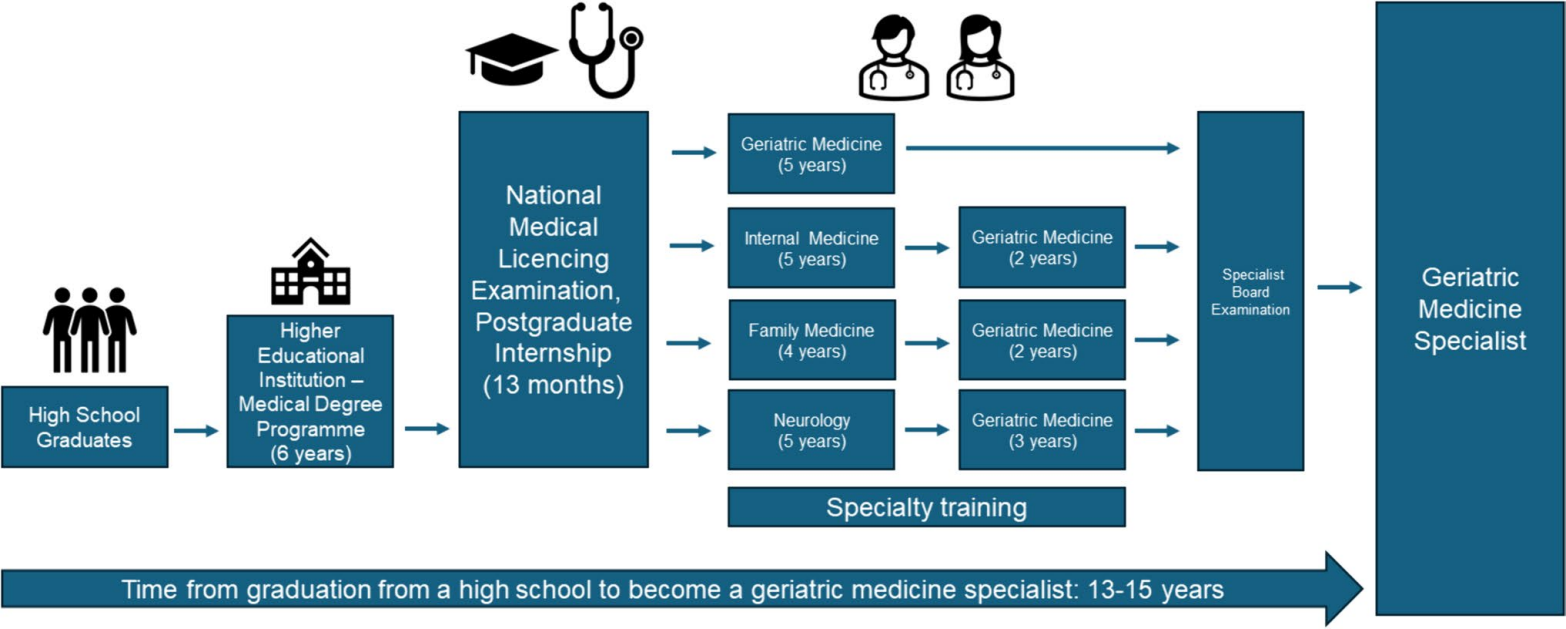
The visual presentation of Polish training required to become a geriatric medicine specialist

The shortage of staff is one of the most demanding challenges that the Polish healthcare system is facing. At present, there are 76 different medical specialties recognised in Poland. According to the latest data provided by the Supreme Medical Chamber (as of 31st December 2023), there are 581 geriatricians in Poland, among which 569 are professionally active [8]. According to demographic estimates in 2060, the percentage of Poles aged 65 + years or more will reach almost one-third of the whole population of 30.9 mln [9]. Hence, all medical students should have the opportunity to complete the learning objectives that would allow them to deal with the medical demands of a growing geriatric population. On the other hand, those choosing geriatric medicine as their professional career need at least 13 years to become a specialist. The available pathways to become a geriatrician in Poland are presented in Fig. 1.

The aim of the study was to perform a deep analysis of the geriatric undergraduate curricula of Polish HEIs.

## Methods

We analysed geriatrics education courses for MD programmes from all Polish HEIs where at least one education cycle had been completed, i.e. acquisition of MD, and thus we excluded HEIs whose students have not graduated yet. Educational institutions are obliged to publish curricula of the programmes they offer. We have used the curriculum for the 2021/2022 academic year. Once accepted, the programme cannot be amended during its duration. We searched through official websites and the Bulletins of Public Information (*Biuletyn Informacji Publicznej—*BIP) of each institution for information regarding the programme and curriculum for teaching of geriatric medicine. Simultaneously, we approached HEI course coordinators and directors, and when not available, representatives from the offices of the Deans of Medicine to provide us with the most current information (*n* = 11/19). One researcher (RK) analysed all documents obtained, searching for information such as: topics covered and assigned learning objectives, the number of teaching and self-studying hours, assigned ECTS points and recommended literature. Next, we used the currently endorsed 2019 ES for medical programmes to select learning objectives related to geriatric medicine. One researcher (RK) searched the whole document manually and then two authors (KP, JG) searched the document using phrases “*star”, “podeszł *” and “*geriatr *” (stems of Polish words related to ageing and geriatrics). We used those learning objectives as references for HEI curricula and then checked how many of those learning objectives were found in each curriculum, and then conversely how many of the learning objectives included in each curriculum were not strictly related to geriatric medicine (e.g. general medical skills). Furthermore, we performed a literature review looking for learning objectives from the international undergraduate geriatric’s curricula and compared them with learning objectives listed in the Polish curricula.

## Results

As of the academic year 2023/2024, there were 38 HEIs offering medical programmes. Nineteen (50.0%) have conferred a medical degree and those institutions were included in our study. Geriatric medicine is taught at all included HEIs. MDs were launched before 2015 in the majority (*n* = 12) of the analysed HEIs. Nine of them are public medical universities, and nine are public universities with a faculty of medicine. Only one HEI is a private institution with a faculty of medicine. The number of graduates per year varied substantially and ranged between 44 and 590 [10]. Detailed information regarding geriatric medicine teaching at included HEIs is shown in Table 1 more complex information is presented in supplementary materials Information regarding geriatric medicine educational programmes was obtained either directly from course coordinators or the offices of the Dean of Medicine (*n* = 10, 52.6%), or were found on the official website or BIP of an institution (*n* = 8, 42.1%). Data were not obtained for one HEI.

**Table 1:** Detailed information regarding geriatric medicine teaching at included Polish higher educational institutions

HEIs	Established	Year	Total number of contact hours	Lectures	Seminars	Practical classes	e-learning	Simulations	Total number of contactless hours	Consults	Self-learning	Total number of hours	ECTS	Number of assigned learning objectives (geriatrics one)	Number of texts (mandatory/additional)^a^
A	Before 2015	5	35.0	12.0	3.0	20.0	–	–	10.0	–	10.0	45.0	1.5	22(6)	8(5/3)
B	Before 2015	5	24.0	8.0	6.0	10.0	–	-	10.5	1.5	9.0	34.5	1.5	14(9)	N/A
C	Before 2015	5	30.0	–	10.0	10.0	–	10.0	–	–	–	30.0	2.0	N/A	6(4/2)
D	Before 2015	5	30.0	N/A	N/A	N/A	N/A	N/A	–	N/A	N/A	30.0	2.0	31(6)	N/A
E	Before 2015	5	50.0	–	17.0	33.0	–	–	40.0	–	40.0	90.0	3.0	13(7)	6(3/3)
F	Before 2015	4	15.0	5.0	6.0	4.0	–	–	15.0	–	15.0	30.0	1.0	7(5)	2(2/0)
G	Before 2015	4	11.0	–	2.0	3.0	6.0	–	–	N/A	N/A	11.0	1.0	N/A	2(2/0)
H	Before 2015	4	30.0	6.0	6.0	18.0	–	–	30.0	–	30.0	60.0	2.0	14	4(3/1)
I	Before 2015	4	18.0	2.0	4.0	12.0	–	-	-	N/A	N/A	18.0	1.0	13(6)	5(3/2)
J	Before 2015	5	20.0	–	2.0	10.0	8.0	–	–	–	–	20.0	1.0	35(8)	4(2/2)
K	Before 2015	5	40.0	10.0	–	30.0	–	–	15.0	–	15.0	55.0	2.0	16(6)	5(3/2)
L	Before 2015	4	30.0	8.0	10.0	12.0	–	–	–	N/A	N/A	30.0	2.0	30(7)	4(3/1)
M	2015	5	50.0	15.0	20.0	15.0	–	–	25.0	-	25.0	75.0	3.0	N/A	5(2/3)
N	2015	5	20.0	10.0	10.0	–	–	–	–	N/A	N/A	20.0	1.0	N/A	6(2/4)
O	2015	5	30.0	10.0	5.0	15.0	–	–	–	N/A	N/A	30.0	1.0	9(5)	5(3/2)
P	2016	N/A	45.0	10.0	15.0	20.0	–	–	35.0	–	35.0	80.0	3.0	6	3(2/1)
Q	2017	4	42.0	12.0	12.0	12.0	–	6.0	48.0	3.0	45.0	90.0	3.0	7(5)	4(1/3)
R	2017	5	60.0	15.0	15.0	30.0	–	–	60.0	10.0	50.0	120.0	4.0	11(5)	3(2/1)

*N/A* not available, information not mentioned, if marked as “–” such a learning method was not included

^a^Mandatory—positions students are expected to use during the course, additional—positions students are suggested to use to widen their knowledge in the field

The curricula were heterogeneous in terms of structure and content. One HEI conducted geriatrics together with palliative medicine (5.3%). All analysed curricula (*n* = 18) contained information including the name of the course, the academic year that the course is assigned to, the total number of course hours and ECTS points. The division into specific teaching forms was found in 17 cases (94.4%), and estimated number of self-learning hours in 10 (55.6%). In 16 instances (88.9%), course contents were described with learning objectives derived from the ES, whereas 2 HEIs presented self-described requirements or teaching content. In one setting, information on the type of formal final assessment was not available.

Each course consisted of a different number of total contact hours (min–max: 11–60, median 30, Q1–Q3: 20–42). Passive teaching was delivered in the majority of the examined HEIs (*n* = 13, 72.2%) that ranged from 2 to15 lecture hours per course. Seminars were conducted in 16 settings (89%; min–max: 2–20 h/course), practical classes in 17 instances (94.4%, min–max: 3–33 h); e-learning was included as a form of teaching in only two programmes, whereas simulation was included in one programme. In nine cases (47.4%), the majority of the planned teaching was lecture based.

Assigned ECTS points also varied between analysed institutions, with a range of 1–4 points; eight HEIs were valued with less than two ECTS points. In most settings (*n* = 13, 72.2%), students received credits with a grade, including six courses (33.3%) ending with a mandatory examination and seven (38.9%) ending with a grade obtained based on the overall performance. In 22.2% cases, the courses ended only with gained credits.

There were 315 learning objectives in total in the 2019 Polish ES,while there were only 9 learning objectives (2.9%) recognised as related specifically to geriatric medicine by the authors: 7 described knowledge requirements, 2 described skill requirements. We found that all learning objectives classified as related to geriatrics were not always included in all HEIs curricula. The geriatric LOs are shown in Table 2. Some learning objectives included in the ES were crucial in geriatric medicine, but were general and not geriatrics specific.

**Table 2:** Learning objectives referring to geriatrics, extracted from the 2019 Polish educational standards

Learning objective	The frequency of each learning objectives in curricula available (*n* = 18)
Knowledge—the student knows	
B.W23 the mechanisms of organism aging	1
D.W04 social attitudes toward the importance of health, illness, disability, and old age, social consequences of illness and disability, as well as socio-cultural barriers, and the concept of quality of life conditioned by health status	2
E.W08 course and symptoms of the aging process, as well as the principles of comprehensive geriatric assessment and interdisciplinary care in relation to older patients	14
E.W09 causes and basic differences in the most common diseases in older people, as well as principles of management in basic geriatric syndromes	14
E.W10 basic principles of pharmacotherapy for diseases in older patients	14
E.W11 threats associated with the hospitalization of older individuals	14
E.W12 basic principles of organizing care for older individuals and the burdens on the caregiver of an older person	13
The student is able to	
E.U21 recognize situations in which the remaining life expectancy, functional status, or patient preferences limit adherence to guidelines specified for a given disease	9
E.U35 assesses pressure sores and applying appropriate dressings	6

*X.W/U00* X represents the classification from the ES, *W* knowledge, *U* skills, *00* the next number in the order

Among recommended literature, we found 29 texts issued between 1995 and 2021. Each HEI recommended at least two texts (median 4.5, min–max: 2–8), while eight (44.4%) recommended at least five texts. Two out of 29 suggested books were published in English, while the remainder were Polish. Among mostly recommended texts, two were issued in 2016 (found 12 times), one in 2018 (6 times), one in 2006 (6 times), and one in 2020 (5 times).

## Discussion

In our research, we analysed the structure of geriatric medicine courses at the undergraduate level in Polish medical programs offered by various universities and higher education institutions in Poland. To our best knowledge, this is the first study focusing on undergraduate courses in central and eastern European medical education. We found that courses covering geriatric medicine are offered at all HEIs included in the study. However, remarkable differences regarding the curriculum and its content were identified. The courses varied in length and students were expected to dedicate different amounts of time to the topic. The recommended literature mentioned in the documents has been often outdated and there was a low number of non-Polish texts. Based on the analysis, we may assume that students were not expected to use modern sources, databases, or international journals during the courses. Familiarizing oneself with foreign, especially English, nomenclature would be worthwhile, as nowadays global guidelines and novel findings are typically published in English. Despite the diversity of the courses’ characteristics, students are meant to acquire comparable knowledge, skills and competencies.

According to the European Union (EU) regulations, it is mandatory to complete a medical program that consists of at least 5500 h to become a physician [11]. All the EU members are obliged to adjust their national programmes to comply with these regulations. In Poland, the Minister of Science and Higher Education issues the educational standards that describe the teaching process [5–7], using EU documents as the basis. The ES aims to provide clear guidelines for faculty members on how to create a programme, so all graduates obtain a comparable education. Importantly, the ES do not state which teaching modalities should be used, nor do they specify the number of hours to be spent between lectures, seminars, and bedside teaching. However, the general suggestions and requirements regarding teaching staff, infrastructure, and summer clerkships are provided. The main component of the ES are learning objectives. Each graduate with an MD is said to have obtained knowledge, skills and competencies presented in the ES and formulated with the use of learning objectives. In September 2023, the update of the ES was published. Since new ES have been issued, an animated discussion regarding teaching of geriatrics should emerge. The international and European regulations should be adaptable and possible to be implemented into many different systems. In recent years, many new MD programmemes have been launched in several HEIs in Poland. The process started in 2015 when three universities were allowed to start an MD programme. At the time of writing this article, there were 38 institutions which offer the programme, while only 19 HEIs have graduates. The number of enrolled students for MDs in 2023 was twice as large than 10 years prior [12–14]. A similar situation was observed few years ago in Spain, where many new institutions had been starting the MD [15]. So far, we are not able to determine students’ knowledge and skills in geriatric medicine, as there is no official standardized examination that would specifically assess those elements. The national medical licencing examination in Poland (*Lekarski Egzamin Końcowy, LEK)* does not include questions covering geriatrics [16]. While questions referring to older patients or medical conditions that mostly appear in older age may appear, they do not test the knowledge of the concepts of geriatric medicine. What is more, the LEK has been recently widely criticized in the professional press for its structure and diminished role in assessing the graduates’ knowledge after the question bank having been introduced [17, 18].

Each course taken by students should be described in detail in curricula. Based on our research, we found out that curricula are not always publicly available. As external individuals we were not able to confirm whether students have access to that information. Having read the curriculum, a student should know every detail regarding the course. That is why HEI’s authorities or course coordinators should provide students with complete and comprehensive documents. However, there is no national blueprint of the curriculum that would work as an example to use. Having one would minimise the differences between universities and even between courses at the same institution. We demonstrated that not every HEI offered complete documentation, missing elements in various sections concerning recommended readings, assigned learning outcomes or the amount of scheduled teaching hours.

Most courses, especially in medical sciences, consist of lectures, seminars or practical classes organised in various settings, including biochemical laboratories, anatomy laboratories or bedside teaching. Many teaching methods are known worldwide [19, 20]. Although the lecture is still a common method, currently it is considered to be rather old fashioned and is proved to be less effective [21]. Undoubtedly, modern technology and digitalisation have had a significant impact on its position, especially as the COVID-19 pandemic forced significant changes in how teachers and students interact. Problem-based learning, team-based learning, self-directed learning, flipped classroom, simulations, case-based teaching, and many more are well-known teaching methods used at universities across the globe [22]. Likewise, there is still room for some improvement. In terms of geriatrics, some innovative teaching methods have been described worldwide. Gamification was suggested by Schlögl et al. as a method worth using to discuss the aspects of polypharmacy [23]. Bhattacharya et al. proved that interprofessional teaching enables students to understand the principles of teamwork and makes them better prepared for geriatrics care [24]. These examples should provide an impetus to modernise teaching methods and the understanding of learning in general. Blaschke et Al. proved that, despite the reported decrease of bedside teaching, it remains a crucial element of undergraduate teaching and indicated that multiple patients should be included, increasing the chances to examine them and obtain essential skills [25].

Analysed courses consisted of lectures, seminars and practical classes. Seminars are one of the most common and popular teaching methods in Poland. Nowadays, much is being said about active teaching that stimulates students’ inner motivation for self-directed learning. Unfortunately, Polish seminars are mostly expository. From the authors’ perspectives (who are teachers with a variety of experience in teaching in preclinical and clinical settings), seminars are rarely utilised to actively engage students and foster collaboration on a given topic, instead often resembling a lecture. On the other hand, we lack sufficient research in Polish contexts that would support our beliefs. Each HEIs commends different infrastructure; hence, the clinical setting may be diverse, and thus not prepared to facilitate clinical rotations in geriatrics. As of 2021, there were 57 hospital geriatric units in Poland [26], offering 1112 beds for patients [27]. These geriatrics units were not available in every voivodeship, meaning not every HEI was able to conduct the geriatric medicine course in a dedicated hospital setting. In 2023 a 28-bed geriatric hospital department was opened in the last voivodeship. That may result in organisational, resource and teaching challenges, and as a consequence, a deficit in students’ knowledge and skills regarding working with older patients. These non-academic factors are common challenges in many countries in Europe and across the globe. There is an urgent need for the mapping of educational, social and systemic requirements to organise a well-structured, multi-tiered policy on the care of older patients. Taking that into account, the faculty could arrange part of the training in an artificial environment, including simulations into the core of the MD. Guidelines regarding the usage of simulation in medical education were published more than 10 years ago [28]. Since then, it was found only in one curriculum. Simulations are a common teaching technique used across the globe. It provides an opportunity to practise in a safe environment that would provide the chance for students to obtain skills that would be useful in their professional career. In a geriatric’s curriculum, there might be room to use this method, especially by those HEIs that do not have the access to a geriatrics department or hospital unit. For those with adequate resources, high-fidelity simulation in a non-inferior realistic presentation may challenge students with more varied clinical situations than what may possible with bedside teaching. Keeping in mind the characteristics of geriatric patients, simulations and virtual reality may potentially be helpful to underline the most crucial aspects in care for older individuals. Additionally, simulations are safe for real patients, as they are not always involved [29]. Popularisation of teaching with simulated or standardised patients would be a worth-considering challenge in geriatric context. It would provide an opportunity for students to meet an older person in a controlled environment. It would be also beneficial for older individuals who may be involved in the teaching [30].

Students obtain knowledge from many different sources. Nowadays, students move towards modern sources and are more open to self-directed learning. They use open access online sources or pay for access to commercial courses or resources. Nevertheless, students should be recommended a reading list provided by their course coordinator to ensure the validity of sources used for the course. Information regarding the mandatory reading list should be available in the curriculum and students need to be informed about the range of the knowledge they are supposed to obtain. In the twenty-first century, many students have shifted to modern learning environments, using dedicated learning applications, taking notes on tablets and other mobile devices [31]. Students should obtain the ability to freely use their sources and to critically analyse findings in other resources. In Poland, the number of mandatory readings for geriatrics courses varied reaching between 1 and 5. Interestingly (not shown in this study), there was no correlation between the length of the course and the number of texts students should utilise. Recommended texts were published between 1995 and 2021. Only 2 non-polish texts were found. Nowadays, when most research and clinical guidelines are published in English, students should practise their language skills by reading and learning from international sources. This would be in agreement with the learning objectives provided in Polish ES that graduates should be able to use English in their occupational career at B2 level, especially as graduates decide to emigrate to western European countries, including the UK and Ireland [32]. Available research indicates that approximately 7% of graduates in Poland take some steps to migrate after graduation [33], while much more consider migration [34]. Using international sources and non-Polish texts would emphasise the continuous need for professional self-development and encourage young doctors to access the most up-to-date guidelines before they are translated and introduced into the Polish system. What is more, the evolving geopolitical situation in some parts of the globe results in increased migration among healthcare providers who have to have their qualifications recognised, thus enforcing the validity of using English sources to facilitate this process. The lack of electronic sources mentioned in curricula is concerning. Most students are digital native, and materials provided for them should be adjusted to a changing world and adapted to a younger generation’s expectations and aptitudes. Han et al. in their review indicated that student-driven learning enhanced with modern technology would be one of the changes incorporated in future medical education [35].

Geriatric medicine remains an unpopular specialty not only among young Polish doctors, but it is an international difficulty. As indicated by Gurwitz J. et al. [36], the number of geriatricians in the USA has fallen in the last 20 years by almost 3000. Currently, in Poland, there are 565 professionally active geriatricians, resulting in 0.07 geriatricians per 1000 citizens aged over 65 years [37] It is essential then to provide other specialists and health professionals with basic skills and knowledge in geriatric medicine. To do so, it is crucial to establish common curriculum or guidelines regarding training in geriatrics for non-geriatricians. It is the aim of some international projects in Europe such as PROGRAMMING COST Action 21,122 [38]. Nowadays, the geriatrics course is typically organised during the 10th or 11th semester. Students may use their previously obtained abilities to learn about aging and the clinical approach to the older patient and learn about principles that highlight the difference between geriatrics and other non-surgical specialisations. It would be challenging to facilitate a geriatric medicine course earlier, as students should be able to determine and understand the differences in geriatrics from other specialties. On the other hand, a course arranged during one of the later semesters may not be considered interesting or students may not be eager to learn, as they may be burned out or look forward to the end of the studies [39, 40].

In September 2023, the ES was updated, and a new version was issued. Having compared them with the previous version, we find many similarities—both focus on the multidisciplinary needs of older persons, aim to furnish students with knowledge, skills and competences on dealing with the most common medical conditions among older patients, understanding of the distinctiveness of geriatric medicine and the difficulties facing older patients and their families and addressing the burden of their medical conditions. All HEIs need to adapt and include the updated ES to their programmes, meaning that whole new curricula have to be prepared. The autonomy enables each institution to arrange the teaching not only according to their preferences and capabilities, but also within their limitations. To address this, lawmakers introduced some amendments, for example, broadening the range of personnel eligible to conduct courses.

The analyses of undergraduate geriatric medicine education at the national level, including Germany, Austria and the UK have been carried out [41–43]. In regard to utilizing the teaching across Europe, a complex proposal of the European curriculum was presented in 2014 by Masud et al. [44]. The proposal was an international consensus of national experts from 29 countries. The authors extracted ten main domains that are necessary for future doctors, after three modified Delphi rounds. If we compare it with the Polish ES and the curricula analysed in this manuscript, we can find some similarities. However, the range of geriatric topics explicitly included in the Polish documents is narrower than in the European ones. As there is a shortage of geriatricians in Poland, many aspects of geriatric medicine are taught not only by geriatricians, but also by other specialists, e.g. neurologists (dementia, degenerative diseases), psychiatrists (depression and other mood disorders, delirium), rheumatologists (osteoporosis) and others. However, there are numerous challenges to overcome. First of all, other specialists may not be able to explain these aspects from a geriatric perspective, and some crucial details may be missed or presented differently, which can be a source of inequalities and deepen the heterogeneity in teaching. The European curriculum provides a complete basis to create a national curriculum adjusted to the contexts of the local healthcare and higher education systems. The Masud’s curriculum describes the content of teaching but not the methods, which enables for implementation of the curriculum in various settings. It presents the complexity of geriatric medicine, underlining not only medical issues (pathophysiology of ageing, atypical presentation of diseases in older age, basis of pharmacology and deprescribing) and aspects of professionalism (physical examination techniques, history taking, distinctness of patient–doctor communication), but also ethical and legal aspects of care of older patient as well. Countries where geriatric medicine is still underdeveloped or not even recognised as a specialty can benefit from these examples when implementing their own programmes. Our example of Polish heterogeneity in geriatric medicine courses across the country underlines the need for a harmonised and well-thought-out plan. Given the diversity of national systems and solutions, there is a need for a rigorous assessment of national curricula or other documents describing undergraduate medical training in geriatric medicine. Once national needs have been identified, further work is needed to harmonise training. With that in mind, the PROGRAMMING COST Action 21,122 has been taking the first steps to prepare a common standardised curriculum to improve the care of older patients.

In terms of the teaching techniques, Masud et al. in their scoping review [45] recommended various methods, including reflecting writing, student journaling, simulations, and e-learning. According to the authors, the teaching should be held in numerous settings, including nursing homes. What is more, vertical integration is highly recommended; students should be exposed to the same aspects of geriatrics from the early stages of their training. Some underline that vertical integration, when implemented properly, affects students’ motivation positively, engaging them for further studies [46]. Recently, entrustable professional activities (EPAs) have been transferring from postgraduate to undergraduate medical education. Some competency-based curricula have already been created. von Streng Paats et al. have concluded that geriatric learning objectives might be described using an EPA framework [47]. Geriatrics remains one of the most actively evolving medical specialties The teaching process should catch up and navigate the discipline for further development [48].

## Conclusions

Geriatric medicine is one of the mandatory courses for medical students in Poland. Polish geriatric courses are heterogeneous in terms of all analysed aspects. The courses vary and are organised in different ways, while students are meant to acquire comparable skills. Demographic predictions together with a challenging workforce situation in many European (including Polish) healthcare systems, including lack of interest among young Polish doctors in pursuing a career in geriatric medicine, underline the urgent need to implement a common undergraduate curriculum in geriatrics. It should enable medical students to gain essential knowledge, skills and competences to work with older patients, regardless of their career choice. We showed that in geriatric medicine there is still room for improvement in terms of teaching methods used to conduct the geriatrics course. This study may be used as an incentive for researchers from other countries to perform a similar analysis of their local programmes. More attention should be paid, both at the national and the European levels, to harmonize the curricula to activate students and to show the wide range of geriatric problems in varied settings.

## Supplementary Information

Below is the link to the electronic supplementary material.

Supplementary file1 (DOCX 40 KB)
