# Supplementary material for: Is geriatric medicine teaching homogeneous? The analysis of geriatric medicine courses at Polish undergraduate medical programmes
Source: Eur Geriatr Med. 2024 Jun 19;15(5):1523–32. doi: 10.1007/s41999-024-01004-y (PMC11614939; doi:10.1007/s41999-024-01004-y)
Supplement: Supplementary file 1 — Supplementary file1 (DOCX 40 KB) [file 41999_2024_1004_MOESM1_ESM.docx]

Supplementary materials

Table 1. Detailed information regarding geriatric medicine teaching at included Polish higher educational institutions.

| HEIs | Established | Type (public (pc)/private (pt)) | Number of graduates* | Chair of Geriatrics/Department | Year/ | Type of examination (exam. e/credits with mark. cm/credits. c) | Total number of contact hours | Lectures | Seminars | Practical classes | e-learning | Simulations | total number of contactless hours | Consults | Self-learning | Total number of hours | ECTS | Number of assigned learning objectives (geriatrics one) | Number of recommended texts (mandatory) | Number of recommended texts (additional) | Total number of recommended texts |
| --- | --- | --- | --- | --- | --- | --- | --- | --- | --- | --- | --- | --- | --- | --- | --- | --- | --- | --- | --- | --- | --- |
| A | before 2015 | pc | 174 | + | 5 | N/A | 35.0 | 12.0 | 3.0 | 20.0 | - | - | 10.0 | - | 10.0 | 45.0 | 1.5 | 22(6) | 5 | 3 | 8 |
| B | before 2015 | pc | 175 | + | 5 | CM | 24.0 | 8.0 | 6.0 | 10.0 | - | - | 10.5 | 1.5 | 9.0 | 34.5 | 1.5 | 14(9) | N/A | N/A | N/A |
| C | before 2015 | pc | 250 | +/- | 5 | E | 30.0 | - | 10.0 | 10.0 | - | 10.0 | - | - | - | 30.0 | 2.0 | N/A | 4 | 2 | 6 |
| D | before 2015 | pc | 590 | +/- | 5 | C | 30.0 | N/A | N/A | N/A | N/A | N/A | - | N/A | N/A | 30.0 | 2.0 | 31(6) | N/A | N/A | N/A |
| E | before 2015 | pc | 250 | +/- | 5 | E | 50.0 | - | 17.0 | 33.0 | - | - | 40.0 | - | 40.0 | 90.0 | 3.0 | 13(7) | 3 | 3 | 6 |
| F | before 2015 | pc | 308 | - | 4 | CM | 15.0 | 5.0 | 6.0 | 4.0 | - | - | 15.0 | - | 15.0 | 30.0 | 1.0 | 7(5) | 2 | 0 | 2 |
| G | before 2015 | pc | 529 | + | 4 | C | 11.0 | - | 2.0 | 3.0 | 6.0 | - | - | N/A | N/A | 11.0 | 1.0 | N/A | 2 | 0 | 2 |
| H | before 2015 | pc | 264 | - | 4 | C | 30.0 | 6.0 | 6.0 | 18.0 | - | - | 30.0 | - | 30.0 | 60.0 | 2.0 | 14 | 3 | 1 | 4 |
| I | before 2015 | pc | 157 | +/- | 4 | CM | 18.0 | 2.0 | 4.0 | 12.0 | - | - | - | N/A | N/A | 18.0 | 1.0 | 13(6) | 3 | 2 | 5 |
| J | before 2015 | pc | 584 | + | 5 | C | 20.0 | - | 2.0 | 10.0 | 8.0 | - | - | - | - | 20.0 | 1.0 | 35(8) | 2 | 2 | 4 |
| K | before 2015 | pc | 343 | + | 5 | CM | 40.0 | 10.0 | - | 30.0 | - | - | 15.0 | - | 15.0 | 55.0 | 2.0 | 16(6) | 3 | 2 | 5 |
| L | before 2015 | pc | 44 | - | 4 | E | 30.0 | 8.0 | 10.0 | 12.0 | - | - | - | N/A | N/A | 30.0 | 2.0 | 30(7) | 3 | 1 | 4 |
| M | 2015 | pc | N/A | N/A | 5 | CM | 50.0 | 15.0 | 20.0 | 15.0 | - | - | 25.0 | - | 25.0 | 75.0 | 3.0 | N/A | 2 | 3 | 5 |
| N | 2015 | pc | N/A | + | 5 | CM | 20.0 | 10.0 | 10.0 | - | - | - | - | N/A | N/A | 20.0 | 1.0 | N/A | 2 | 4 | 6 |
| O | 2015 | pc | N/A | - | 5 | CM | 30.0 | 10.0 | 5.0 | 15.0 | - | - | - | N/A | N/A | 30.0 | 1.0 | 9(5) | 3 | 2 | 5 |
| P | 2016 | pt | N/A | +/- | N/A | E | 45.0 | 10.0 | 15.0 | 20.0 | - | - | 35.0 | - | 35.0 | 80.0 | 3.0 | 6 | 2 | 1 | 3 |
| Q | 2017 | pc | N/A | + | 4 | E | 42.0 | 12.0 | 12.0 | 12.0 | - | 6.0 | 48.0 | 3.0 | 45.0 | 90.0 | 3.0 | 7(5) | 1 | 3 | 4 |
| R | 2017 | pc | N/A | N/A | 5 | E | 60.0 | 15.0 | 15.0 | 30.0 | - | - | 60.0 | 10.0 | 50.0 | 120.0 | 4.0 | 11(5) | 2 | 1 | 3 |

*data regarding the number of graduate were available in period 2014-2020; + only geriatrics department. +/- department of geriatrics and other medical specialty. - department of other specialty; N/A – not available. information not mentioned;  if marked as “-“ such a learning method was not included.

The links to the official websites of the included institutions:

1. Medical University of Białystok – <https://www.umb.edu.pl/en/index.php>
2. Nicolaus Copernicus University Medical College in Bydgoszcz - <https://en.cm.umk.pl/>
3. Medical Univeristy of Gdańsk - <https://mug.edu.pl/>
4. Jan Kochanowski University of Kielce - <https://en.ujk.edu.pl/>
5. Jagiellonian University Medical College in Kraków - <https://cm-uj.krakow.pl/index.php/en>
6. Andrzej Frycz Modrzewski Krakow University - <https://en.ka.edu.pl>
7. Medical University in Lublin - <https://umlub.pl/en/>
8. Univeristy of Warmia and Mazury in Olsztyn - <https://uwm.edu.pl/en>
9. Medical Univeristy of Lodz - <https://en.umed.pl/>
10. Poznan University of Medical Sciences - <https://pums.ump.edu.pl/>
11. Casimir Pulaski Radom University - <https://uniwersytetradom.pl/?lang=en>
12. University of Opole - <https://www.uni.opole.pl/en>
13. Medical University of Silesia - <https://smk.sum.edu.pl/>
14. Pomeranian Medical University in Szczecin - <https://www.pum.edu.pl/>
15. University of Zielona Góra - <https://uz.zgora.pl/en/>
16. University of Rzeszów - <https://www.ur.edu.pl/en/home>
17. Medical University of Warsaw <https://www.wum.edu.pl/en>
18. Wrocław Medical University - <https://www.umw.edu.pl/en/contact-umw>

Table 2. Distribution of the learning outcomes by course groups with the minimum number of hours and ECTS points.

| Course Groups | Number of Hours | ECTS Points |
| --- | --- | --- |
| A. Morphological Sciences | 300 | 25 |
| B. Scientific Foundations of Medicine | 525 | 43 |
| C. Preclinical Sciences | 525 | 43 |
| D. Behavioral and Social Sciences with Elements of Professionalism | 240 | 12 |
| E. Non-surgical Clinical Sciences | 1060 | 65 |
| F. Surgical Clinical Sciences | 900 | 50 |
| G. Legal and Organizational Aspects of Medicine | 100 | 6 |
| H. Clinical Practical Teaching (30 weeks) and Exams | 900 | 60 |
| I. Professional Internships (20 weeks) | 600 | 20 |
| Total | 5150 | 324 |

ECTS - European Credit Transfer and Accumulation System; Each institution possesses additional 550h for their own usage
